# “Alexa, What’s a Phishing Email?”: Training users to spot phishing emails using a voice assistant

**DOI:** 10.1186/s13635-022-00133-w

**Published:** 2022-11-22

**Authors:** Filipo Sharevski, Peter Jachim

**Affiliations:** School of Computing, DePaul University, Chicago, IL USA

**Keywords:** Phishing training, Voice assistants, Alexa

## Abstract

This paper reports the findings from an empirical study investigating the effectiveness of using intelligent voice assistants, Amazon Alexa in our case, to deliver a phishing training to users. Because intelligent voice assistants can hardly utilize visual cues but provide for convenient interaction with users, we developed an *interaction-based phishing training* focused on the principles of persuasion with examples on how to look for them in phishing emails. To test the effectiveness of this training, we conducted a between-subject study where 120 participants were randomly assigned in three groups: no training, interaction-based training with Alexa, and a facts-and-advice training and assessed a vignette of 28 emails. The results show that the participants in the interaction-based group statistically outperformed the others when detecting phishing emails that employed the following persuasion principles (and/or combinations of): authority, authority/scarcity, commitment, commitment/liking, and scarcity/liking. The paper discusses the implication of this result for future phishing training and anti-phishing efforts.

## Introduction

The advances in filtering phishing emails/messages [1] and usable security warnings [2] still leave a considerable room for phishers to successfully exploit victims. The last line of phishing defense, thus, is a phishing awareness training. If we can train users to spot a phishy URL or an overtly influencing and persuasive email narrative, the chances are that the phishers will be left empty handed [3]. The common anti-phishing advice is to “hover over links to check their URLs and where the URLs lead to” or “not to open a suspicious attachment” [4]. The phishing awareness training comes in various formats. Users have the option to read facts-and-advice materials [5], participate in a phishing game [6], role-play in a simulated phishing scenario [7], or use an app to practice fraudulent URL detection [8]. The phishing awareness training sometimes is taken further and users are shown the real-world consequences of following a phishing URL or attempting to induce high-fear situations [9]. In most cases, the exposure to phishing training resulted in improved levels of spotting phishing emails and decreased susceptibility, proving that is an effective strategy in combating persistent phishers.

A characteristic and critical component for the success of all of the aforementioned phishing awareness trainings is that the phishing emails are demonstrated using visual cues, that is, users are given examples of known phishing emails, URLs, and attachment and carefully explained the differences between them and their legitimate counterparts. This modality is preferred because it is assumed that users mostly rely on *visual* inspection of the email through a screen, either a personal computer, laptop, tablet, or a smartphone. While the visually based phishing awareness training has received a tremendous research attention, very little is known about the effectiveness of a phishing awareness training delivered through an interaction with an intelligent voice assistant like Amazon Alexa or Google Home.

Amazon and Google allow for customization and use of third-party apps, called “skills” or “actions,” respectively. The skills and actions, like browser extensions, offer a variety of services Alexa or Home themselves do not provide [10]. A recent study explored how an attacker can implement a malicious logic in a skill to manipulate news briefings in order to induce misperception [11]. The results suggest that by manipulating the email text, a skill could influence users to misperceive phishing emails as legitimate ones. These results prompted us to explore how skills can be used to combat such phishing misperceptions. For this study, therefore, we developed an Amazon skill that delivers a phishing awareness training through a voice-based interaction with a user.

To test the effectiveness of the phishing awareness training skill, we conducted a between-subject study where 120 participants were randomly assigned in three groups: (1) no training, (2) interaction-based training with Alexa, and (3) facts-and-advice training. Each of the participants, after receiving a training (or simply proceeding), was given a vignette of 28 emails and assessed whether each one of them was phishing or not. Participants were also asked to elaborate on the cues they used to make their decision. We also measured participants’ “phishing awareness” using the SeBIS scale [2] and used the scores together with their responses to determine the immediate effect of the phishing training delivered though interaction with Alexa.

With this work, we built an alternative method of delivering a phishing awareness training—interaction with Alexa—to the traditional training methods. Our goal is to understand how the concept of phishing materializes within an intelligent voice assistant environments based on the users’ trust in these devices. This paper proceeds as follows: Section 2 reviews the current landscape of phishing awareness training methods, Section 3 contextualizes the phishing awareness training in an intelligent voice assistant environment and describes the concepts of *interaction-based phishing training*, Section 4 details the study design, and Section 5 reports the study results. The implications of the results for raising phishing awareness are discussed in Section 6 together with a proposal for phishing warnings and training bundle of skills for intelligent voice assistants users. Finally, Section 7 concludes the paper.

## Phishing awareness training

Users are independent agents that make their own decisions, but the majority are not experts in computers nor are aware of the phishing threat. The reasonable strategy, then, is to persuade the users to change their security behavior when deciding on emails and phishing websites [5]. The most common method of changing user’s security behaviors is phishing awareness training. Users in these phishing awareness trainings are either given facts-and-advice materials [4], exposed to phishing stories, [5, 12], participate in a game [6], role-play in a simulated phishing scenario [3, 7], or use an app to practice fraudulent URL detection [8]. In this section, we review each of these phishing training methods with their advantages and disadvantages to inform the best approach for creating an interactive-based phishing training.

### Facts-and-advice

Facts-and-advice materials explain phishing and how not to fall for it from an authoritative perspective of a security expert. These materials fit a common pattern, providing definition of phishing and generic examples of harm (decontextualized factual information) followed by a generic advice in the form of 2^nd^ person imperative statements (“you should do X” or “don’t do X”). For example, a common advice is to “hover over links to check their URLs and where the URLs lead to” or “Be wary of messages demanding immediate response and requesting passwords, bank accounts, or threatening to suspend or terminate your account” [4, 5].

The facts-and-advice are shown to have positive effect in increasing phishing awareness, but the shortcomings of this approach is that they are often incomplete from a user’s point of view [13]. For example, many people take this advice but might not be prolific in understanding URLs, especially ones that are obfuscated in tiny forms. Facts-and-advice training does not contain information about who might be the phishers, what approaches they use to influence victims, or the detailed social and personal consequences of actual phishing attacks. Nonetheless, facts-and-advice training is suitable for delivery through an intelligent voice assistant because the advice comes from a “trusted voice” and could be delivered on-demand in people’s home environments.

### Stories

Stories about experiencing phishing predicaments frame the phishing awareness training as a sequence of actions rather than as factual statements in order to help users learn from experience.. The phishing stories convey lessons about how to behave in various circumstances when deciding about a suspicious email [14]. A study comparing traditional facts-and-advice training against training that uses a phishing story found that the facts-and-advice training works better when presented by a security expert, and the stories work much better when told by a peer [5]. The use of stories for phishing awareness training highlights the need to consider both the perceived authority of the “trainer” (origin of training materials) as well as the relevance of the story to the user [12]. Alexa, in this case, could serve as a trusted authority, given that Alexa already serves as an “assistant” for delivering information relevant to users [15]. A recent study suggests that users are more likely to consent to share a conversation or listen to a story when they do not find it sensitive with their voice assistants [16].

### Phishing games and simulations

Cybersecurity training and education through gamification is an effective method for chaining security behavior and skills development [17, 18] because users can participate without fear of consequences. Cybersecurity games incentivize and motivate users to engage in an activity that would otherwise not engage, for example, focus on examining a suspicious URL in an email. Additionally, game-based education attracts and retains the user until the end of the game by providing immediate feedback [19]. Leveraging the benefits of game design, authors in [6] developed and evaluated a phishing game to teach users how to identify phishing URLs, where to look for cues in web browsers, and how to use search engines to find legitimate websites. The results of the study show that game-trained users performed better in correctly identifying phishing websites but were no better in incorrectly judging phishing websites to be a legitimate compared to users who were given facts-and-advice or no training at all.

A similar game for mobile platforms was developed in [19]. The evaluation of the mobile phishing game showed that it positively raised users’ motivation to examine suspicious URLs before deciding to proceed with a suspicious email. Authors in [3] introduced a game that simulates actual phishing attacks in a role-playing game to encourage the player to practice defending themselves. The idea was to present phishing awareness information in context in order to better transfer the solving faculties out of the game and in the real life. Although the games show positive effect on raising awareness, at this point, it might be difficult to transfer them straightforwardly into the intelligent voice assistant environment.

### Principles of influence in phishing training

All of the aforementioned training methods are focused on training users to differentiate phishing URL from a legitimate one, look for cues in web browsers, or check for a illegitimate email sender. Modest or no effort is invested in introducing the principles of influence to users and how to look for them, outside of implicit facts-and-advice about suspicious emails demanding “immediate action” [5]. A study investigating the effectiveness of the principles of influence in phishing context found that users are highly susceptible to emails employing the *authority* principle (e.g., an email sent from an authoritative figure) and to a lesser degree to the *liking* and *social proof* principles [20]. Factoring the demographics of the users, another study found that the younger ones were more susceptible *authority* and *scarcity* but the older ones to the *liking* and *reciprocation* [21]. Additionally, *scarcity* and *reciprocation* are found to successfully influence users to open a phishing website [22].

We choose to implement an interaction-based phishing awareness training that incorporates the principles of influence for several of reasons. First, the principles are general and do not depend on the particular email formatting and known phishing cues used in conventional facts-and-advice or game/simulation trainings. These cues are useful, but could be limited to URL formatting that is abandoned (e.g., replaced with tiny URLs instead) or past or irrelevant graphical/language formatting (e.g., old PayPal or Amazon emails) [5, 23]. Second, the principles of influence can be summarized and conveyed as a simple snippet or a story to the user by a “trusted” trainer, that is, Alexa. Users enjoy interacting with voice assistants because they are “seamless enough to be irresistible” and worry mainly about how their personal data is handled, not what information is delivered to them by these assistants [24]. Since the phishing awareness training is entirely delivered to the users (i.e., users do not share any personal data as part of the training), there is no reason for users to question the trustworthiness of Alexa.

Third, the principle of influence enable users contextualize the training to their personal email communication, instead of visually inspecting emails or reading phishing stories with no relevance to them [25]. Fourth, the principles of influence could be of benefit in raising phishing awareness in situations the training is simply completed for the sake of “compliance” [7]. Many people go thought phishing trainings because they are required to do so and less because they are genuinely interested to do so [26]. Based on these premises, we crafted a blend of a facts-and-advice and story training that presents the principles of influence to users as an alternative phishing awareness training.

## Alexa the phishing teacher

### Intelligent voice assistant environment

**Fig. 1:**
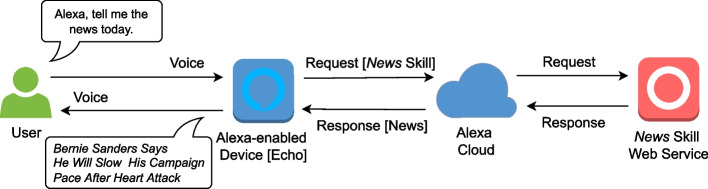
Amazon Alexa intelligent voice assistant environment

The basic elements of the intelligent voice assistant environment are shown in Fig. 1. Amazon introduced voice assistant *skills* to allow Alexa to help users with a multitude of tasks (similar to skills are Google’s *actions*). Skills are essentially third-party apps, like browser extensions, offering a variety of services Alexa itself does not provide [10]. To invoke a skill, the user utters a wake-word, a trigger phrase, and the skill’s invocation name. For example, for the spoken sentence “Alexa, what’s phishing?,” “Alexa” is the wake-word, “what’s” is the trigger phrase, and “phishing” is the skill invocation name. In response, Amazon’s cloud relays this request to the third-party server that returns text content as a result, e.g., “Phishing is an attempt by an attacker to solicit personal information from unsuspecting users.” This response is converted to speech by Alexa and spoken back to the user through the Alexa-enabled device (a similar invocation logic is followed in the case of Google’s actions).

### Interaction-based phishing training

We used the Alexa skill blueprint for Q&A to develop a skill for an interaction-based phishing training. The development process was simple and straightforward and required only customization of the Q&A blueprint to include the training content. The training includes a definition of phishing and interaction flow with Alexa where the trainee first prompts Alexa about definition of phishing emails. Alexa responds with a brief definition of phishing, adapted from [20, 27]. Next, the trainee prompts Alexa to give more information about the principles of influence. Alexa, configured with summarized snippets from [28, 29], responds with brief explanation for each one of them. To help the trainee contextualize the training, the trainee also prompts Alexa for example emails incorporating the principles of influence as shown in Table 1.

**Table 1:** Interaction-based phishing awareness training

User:	Hey Alexa, what’s a phishing email?
Alexa:	Phishing is an attempt by an attacker to solicit personal information from unsuspecting users. To entice users, the attackers usually employ one or multiple persuasion principles when crafting the email such as: authority, commitment, liking, reciprocation, scarcity, and social proof.
User:	Hey Alexa, tell me more about these principles.
Alexa:	The principle of authority states that people comply with requests made by figures of authority, such as doctors or politicians.
	The commitment principle proposes that once a user has taken a stand, they feel pressured to behave consistently with that stance.
	The liking principle states that people tend to comply with requests from people they like, for example friends or celebrities.
	The reciprocation principle states that people tend to repay, in kind, what another person has provided them.
	The scarcity principle states that the perceived value of an offer is increased when its availability is limited.
	The social proof principle states that people act accord to what other people are doing to avoid mistakes.
User:	Hey Alexa, tell me examples of phishing emails that incorporate these persuasion principles.
Alexa:	For example, an email urging a user to download a safety measures document about the COVID-19 pandemic employs the principle of authority, using the World Health Organization as the sender, and the principle of scarcity, using the lack of public knowledge about COVID-19.
	Another example employing the liking/reciprocation principles is an email sent by the Jay Z and Rihanna requesting to match their donation for the COVID-19 pandemic that also offers free tickets to a benefit concert as a reciprocation for clicking on the link included in the email.
	An example of using social proof is an email requesting parents to click on a link and sign a petition to close all schools in the state of New York until a vaccine for the COVID-19 is developed.
	Similarly, the principle of commitment could be incorporated in an email that asks for the recipient to donate money for support of the healthcare workers in New York during the COVID-19 pandemic.

## Study design

Our study investigates the benefits of an interactive-based phishing awareness training, delivered using the Alexa intelligent voice assistant. Because this is a first study of this kind, participants were exposed to (1) no training, (2) facts-and-advice training, and (3) interaction-based training in order to investigate any differences in outcome between these three training methods. The facts-and-advice training first described what a phishing email and next gave the trainees an advice on how persuasion could be used to influence a victim to click on a fraudulent URL.

The interaction-based training was conducted by interacting with Alexa, where Alexa provided answers to the questions in Table 2, that is, Alexa answered what a phishing email is, defined the principles of influence, and described phishing emails incorporating these principles. Participants, following the training (or simply proceeding), were offered a choice to indicate “phishing/no phishing” after each email and answer the open-ended question “‘What cues did you use to decide if this email is phishing or not?”. Participants also completed a SeBIS questionnaire assessing their susceptibility to phishing [2]. Using this data, we tested the following hypotheses:

- **Hypothesis 1:** *There is no difference in detecting phishing emails between the groups exposed to (1) no training, (2) facts-and-advice training, and (3) interaction-based training when compared for individual emails implementing one or a combination of two persuasion principles.*
- **Hypothesis 2:** *There is no difference in susceptibility to phishing, measured with the SeBIS scale, between the groups exposed to (1) no training, (2) facts-and-advice training, (3) interaction-based training when compared for individual emails implementing one or a combination of two persuasion principles.*

### Participants

For the study, we recruited participants at least 18 years or older that have interacted with an intelligent voice assistant at least a few times in their lives (e.g., Alexa, Google Home, Siri, etc.). The participants were recruited from a large university participant pool. A sample of 120 participants agreed to be in the study (54 females, 66 males). We randomly assigned the sample to three groups (18 female/22 male participants/group; total 40): (1) a group that did not receive any training, (2) a group that received a regular facts-and-advice training, and (3) a group that received an interaction-based phishing training. To the first group, the study was announced as an “email assessment” that aims to investigate how an individual assess emails from their inbox in the normal course of a day. The second group and third group received additional information that they will be given visual training or interactive information on various types of persuasion in email communication from Alexa. The study was IRB approved; the participants received and electronically signed an informed consent and were given small compensation for their participation.

### Procedure

The participants in each group then assessed a vignette of 28 emails, presented in random order to each participant, to determine if each email is phishing or not and indicate what cues they used to decide upon. Following the selection criteria for phishing susceptibility experimentation provided in [29], the emails were adapted from a corpus of confirmed phishing attacks compiled from four prominent universities [9]. The adaptation was in the context of the world events during the execution of the study and the current trends in phishing campaigns. These emails were selected from a larger group because they met two primary criteria: (1) they attempted to persuade the recipient to perform some action and (2) they clearly contained at least one of the persuasion principles of interest. The emails were coded accordingly using the coding approach in [28, 29]. Fourteen groups of persuasion principles (or combinations of principles) were derived, as shown in Table 2. For each combination, we included one phishing and one legitimate email.

**Table 2:** Phishing email vignette topics

Persuasion principle(s)	Email topic
Authority (A)	Financial Aid Office
Authority / Commitment (A/C)	Office of the Major
Authority / Liking (A/L)	Community Blood Center
Authority / Scarcity (A/S)	Information Services
Commitment (C)	Political Campaign
Commitment / Liking (C/L)	Campaign Donations
Scarcity / Liking (S/L)	Centers for Disease Control (CDC) and a Foundation
Liking (L)	Cellular Service
Scarcity (S)	Online Shopping Refunds
Reciprocation (R)	Survey Participation
Reciprocation / Liking (R/L)	Recruiting
Social Proof (SP)	Alumni Organization
Social Proof / Liking (SP/L)	People for Ethical Treatment of Animals (PETA)
Social Proof / Commitment (SP/C)	Workplace Benefits

After each email, the participants were asked to provide feedback on (1) whether the email is phishing or not and (2) what cues they used to aid their decision. We decided for an email phishing assessment after each question in our initial study because we wanted to preliminary gauge how users will respond to the idea of interaction-based training, and based on that, use the results to improve the Alexa-based training skill by revising how a particular persuasion principle (or a combination of) is incorporated and explained by Alexa. Following the completion of the phishing assessment, the participants completed the SeBIS questionnaire to measure participants’ *phishing awareness* [2].

SeBIS is an instrument scored on a 5-point Likert scale that measures a computer user’s self-reported intent to comply with “good” security practices such as paying attention to contextual phishing cues, e.g., the web browser URL bar or various security iconography. The experiment was conducted entirely online, hosted on Qualtrics survey software. The participants in the interaction-based learning group interacted with the web-based version of Amazon Alexa (due to the COVID-19 pandemic, we were unable to conduct in-person experiments with the real Alexa device. The web-based version, in any case, provides the same functionality as the device).

## Study results

### Hypothesis 1

**Fig. 2:**
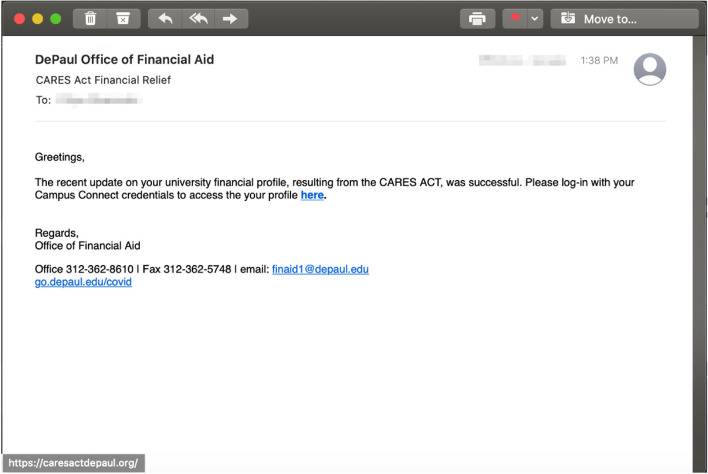
Phishing email incorporating the *authority* principle

The first hypothesis stated that there is no difference in detecting phishing emails between the groups exposed to (1) no training, (2) facts-and-advice training, and (3) interaction-based training delivered through Alexa. We found a statistically significant difference between the groups for *phishing* emails employing the following phishing persuasion principles (or combinations of): authority, authority/scarcity, commitment, commitment/liking, and scarcity/liking, as shown in Table 3. Initially, the interaction-based trained participants outperformed the participants in the other groups by correctly identifying that the email employing the authority principle (Fig. 2) is phishing, $\chi (2) = 7.590$, $p = .022^{*}, (\alpha = 0.05)$.

**Table 3:** Pearson chi-square tests—hypothesis 1

Group	No phishing	Phishing
Authority principle		
No training	16 (40%)	24 (60%)
Facts and advice	12 (32.4%)	25 (67.6%)
Alexa	5 (12.8%)	34 (87.2%)
Authority/scarcity principles		
No training	13 (32.5%)	27 (67.5%)
Facts and advice	11 (28.2%)	28 (71.8%)
Alexa	4 (10%)	36 (90%)
Commitment principle		
No training	17 (43.6%)	22 (56.4%)
Facts and advice	4 (10.3%)	35 (89.7%)
Alexa	3 (7.9%)	35 (92.1%)
Commitment/liking principles		
No training	15 (38.5%)	24 (68.5%)
Facts and advice	13 (33.3%)	26 (66.7%)
Alexa	5 (12.5%)	35 (87.5%)
Scarcity/liking principles		
No training	16 (41%)	23 (59%)
Facts and advice	15 (37.5%)	25 (62.5%)
Alexa	5 (12.8%)	34 (87.2%)

The breakdown in Table 3 does not account to 40 participants per group for all groups because we allowed the participants to skip questions if they feel uncomfortable answering it, per the IRB approval requirements. The email came from the office of Financial Aid Office to indicate an update on the recipient’s financial profile resulting from the CARES Act. The interaction-based trained participants that indicated that “the CARES Act bails local governments, not universities,” and “the greeting is too generic from an authoritative office.” The correct participants in the facts-and-advice group referred to the formatting of the URL which “contained .org instead of .edu domain” as a tip-off cue that the email was phishing.

**Fig. 3:**
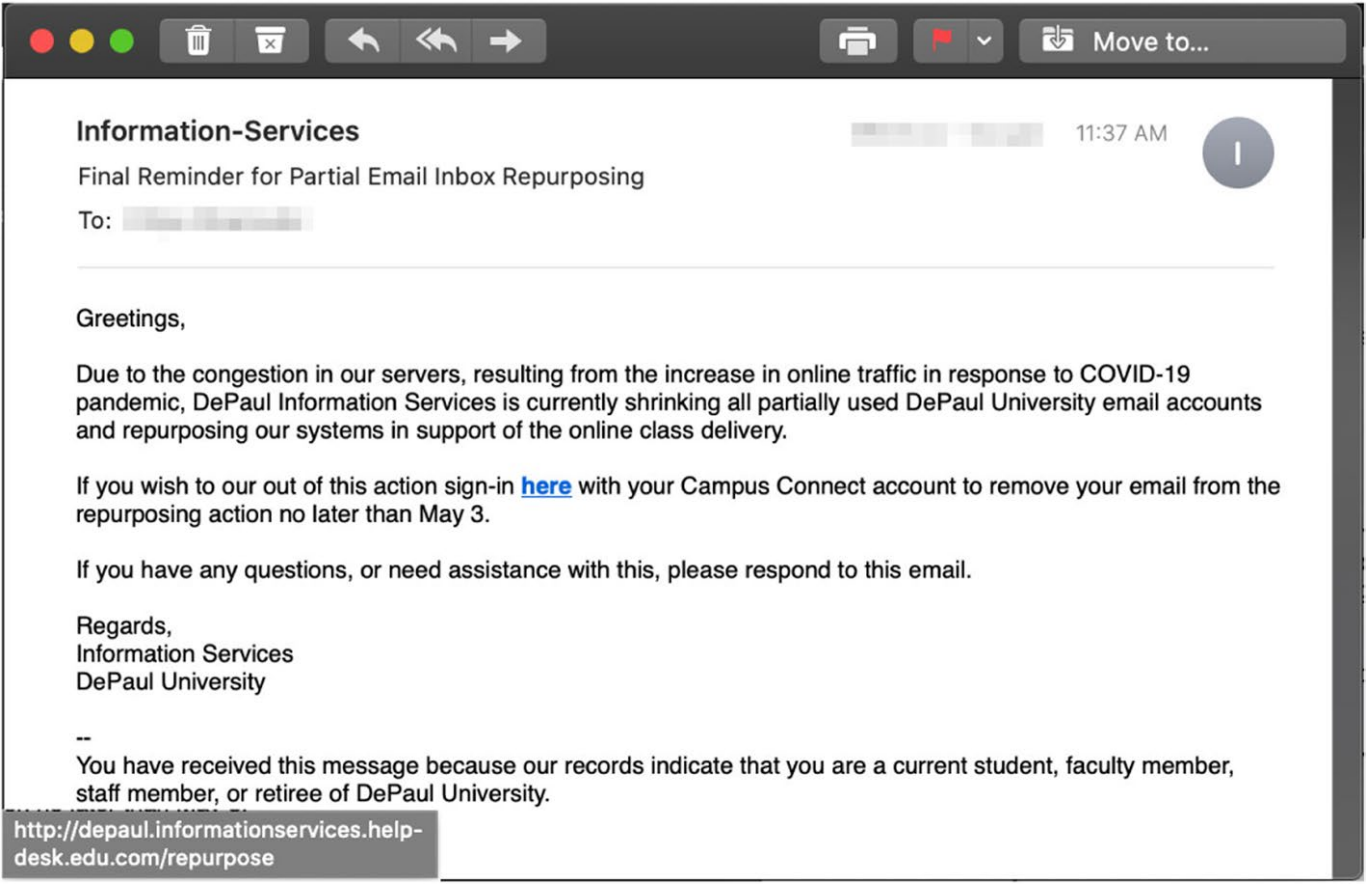
Phishing email incorporating the *authority* and *scarcity* principles

Next, the interaction-based trained participants also outperformed the other participants by identifying correctly that the email employing a combination of the authority and the scarcity principles (Fig. 3) is phishing, $\chi (2) = 6.332$, $p = .042^{*}, (\alpha = 0.05)$ . As in the previous instance, it can be noticed that the facts-and-advice participants performed better than the participants that received no training at all (even with one of the participants in the facts-and-advice group skipping this particular email). In this case, the email came from the IT department, notifying users about a re-purposing of space from unused email accounts and offering an opt-out option from this action. The participants in the interaction-based training group indicated that “it is highly unlikely that any email traffic caused congestion.” The correct participants in the facts-and-advice group mainly referred to the formatting of the email which was missing the usual “logos associated with the administration emails” as a tip-off cue that the email was phishing.

**Fig. 4:**
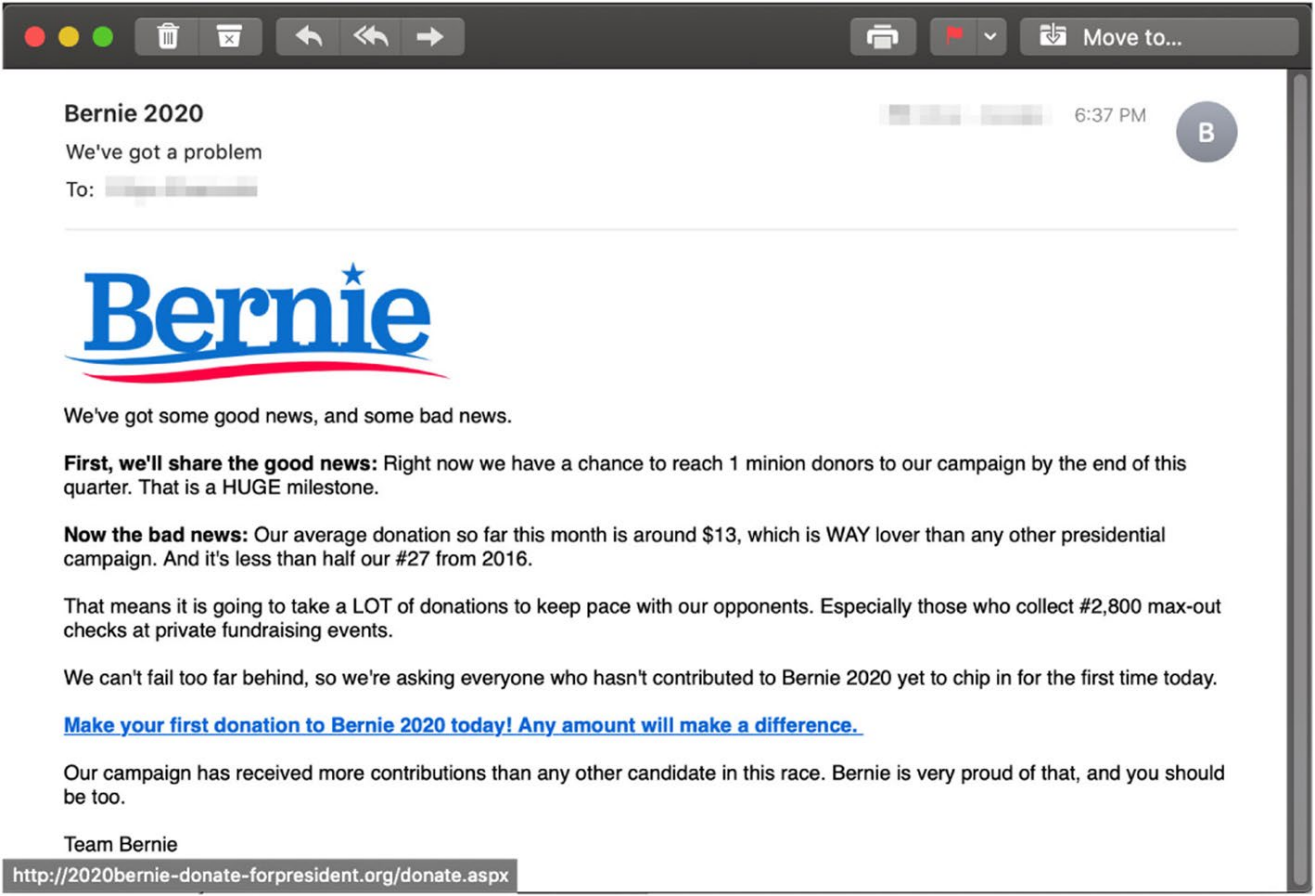
Phishing email incorporating the *commitment* principle

The interaction-based trained participants also outperformed the other participants (sans one response in each group) by identifying correctly that the email employing a commitment principles (Fig. 4) is phishing, $\chi (2) = 18.842$, $p = .000^{*}, (\alpha = 0.05)$. In this case, the email came from a political party campaign, requesting small donation. The participants in the interaction-based training group indicated that “the language in this email doesn’t appear authentic, and one can donate on the candidate website if they want to.” The correct participants in the facts-and-advice group indicated that “the URL is a long and prominent red flag - it’s not the official Bernie site”.

**Fig. 5:**
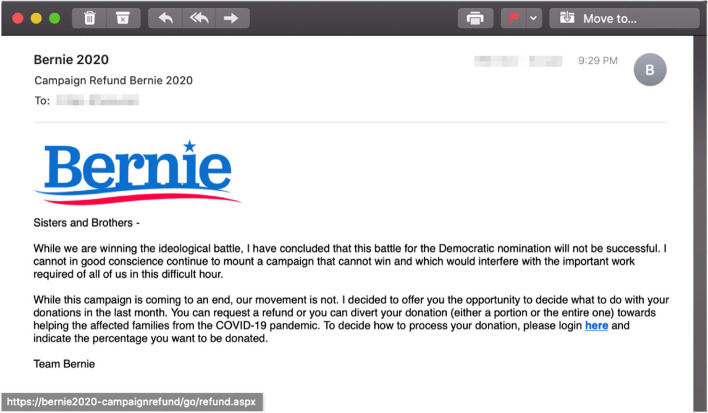
Phishing email incorporating the *commitment* and *liking* principles

The same pattern was observed for the interaction-based trained participants that correctly deemed the email employing a combination of the commitment and the liking principles (Fig. 5) as phishing, $\chi (2) = 7.440$, $p = .024^{*}, (\alpha = 0.05)$. In this case, the email again came from the same political party campaign, offering the recipients a refund of their donations due to conclusion of the campaign. The participants in the interaction-based training group indicated that “donations made to a campaign are final, and it is up to the campaign on how they decide to spend the money - it cannot be given back to users to decide where the money goes.” The correct participants in the facts-and-advice group pointed the URL as a tip-off cue that the email is phishing.

**Fig. 6:**
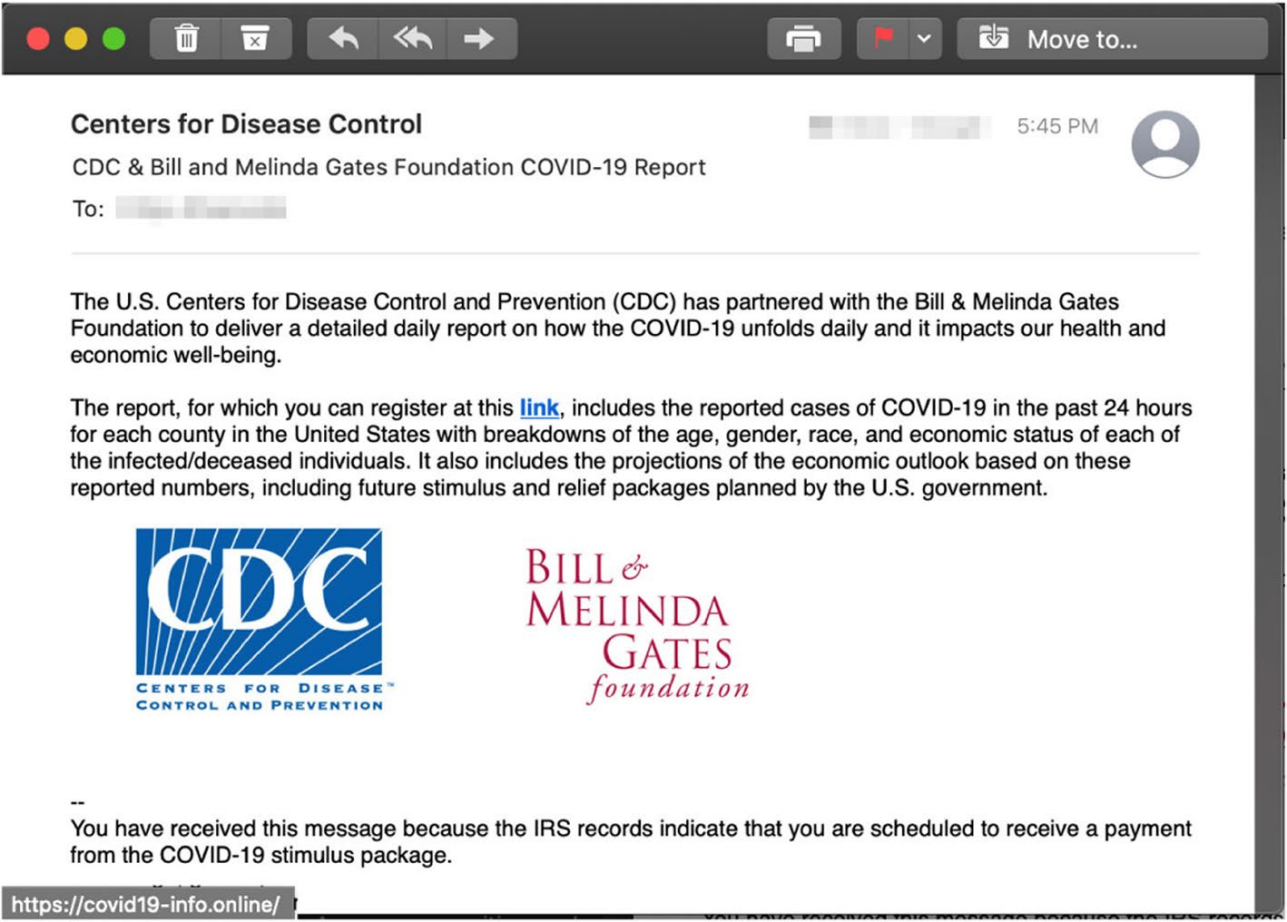
Phishing email incorporating the *scarcity* and *liking* principles

Lastly, the interaction-based trained participants outperformed the other participants and correctly deemed that the email employing a combination of the scarcity and the liking principles (Fig. 6) is phishing, $\chi (2) = 8.712$, $p = .013^{*}, (\alpha = 0.05)$. In this case, the email again came from the Centers for Disease Control (CDC) on the behalf of a large foundation offering the recipients a report on the COVID-19 progress. The participants in the interaction-based training group that correctly deemed the email phishing justified their decision indicating that “CDC is transparent and has all the COVID-19 numbers on their website for free.” The correct participants in the facts-and-advice group again pointed the URL as a tip-off cue that the email is phishing.

In summary, the first hypothesis is rejected for five out of 14 *phishing* emails and the alternative hypothesis accepted. The interaction-based training proved on par with the facts-and-advice training when the emails incorporated the authority, authority/scarcity, commitment, commitment/liking, and scarcity/liking principles of influence. A difference was not observed for the emails formatted with the authority/commitment, authority/liking, liking, scarcity, reciprocation, reciprocation/liking, social proof, social proof/liking, and social proof/commitment. Previous evidence suggests that victims are significantly more likely to click on links in emails using authority and scarcity than those using the other principles of influence [20, 22]. Respectively, the interaction-based training, as the results from our study suggest, has the potential to counter this effect and help users recognize emerging phishing campaigns.

Expectedly, we did not get any significant results for the 14 *legitimate* emails. Upon inspection of the reported cues in determining whether an email is phishing or not, participants in each group were overly suspicious and falsely determined the email as phishing. For example, one participant from the interaction-based group deemed the legitimate email incorporating the authority/liking principle as “phishing” believing that “the email comes from the office of the mayor, which is used as the ‘authority’ hence it is phishing.”

In several other examples, participants expected that the emails incorporating the reciprocation or liking principles should be “directly referenced to them by name instead of being general to show that the sender really has honest intentions and deserves ‘reciprocation’ and/or ‘liking’.” These comments, though without a statistical support, nonetheless warrant attention when proceeding with interaction-based trainings. Users certainly can be overly cautious and security minded [30] but the interaction-based training must stress during the training that legitimate emails also come from authoritative senders and implicitly incorporate the principles of influence. These findings, hence, are useful for our future work and we plan to evolve and test the interaction-based training with respective examples of contrasting legitimate emails.

### Hypothesis 2

The second hypothesis states that there is no difference in susceptibility to phishing, measured with the SeBIS scale, between the groups exposed to (1) no training, (2) facts-and-advice training, and (3) interaction-based training. We did not find a statistical difference between the three groups on the SeBIS scale ($\chi (2) = 3.069$, $p = .216, (\alpha = 0.05)$) so we retain the hypothesis 2. However, the interaction-based training participants ($SeBiS = 3.999$, $\sigma = .62092$) and the facts-and-advice participants ($SeBiS = 4.02$, $\sigma = .70718$) had a higher on average SeBIS score compared to the participants that received no training at all ($SeBiS = 3.7638$, $\sigma = .73641$). We believe that with a larger sample a significance will be achieved, confirming that the interaction-based training as proposed in this paper is on par with the regular facts-and-advice training and certainly better than a no training at all. A further investigation could contextualize the SeBIS scale regarding the principles of influence and compare the susceptibility to phishing prior and post administering the interaction-based and the facts-and-advice training.

## Discussion

Inspired by the rapid proliferation of intelligent voice assistants like Amazon Alexa or Google Home, we investigated how they can be utilized in training users to spot phishing emails. Our analysis shows that the participants who received interaction-based training on the principles of influence through Alexa performed significantly better when detecting five out of the 14 phishing emails in the vignette we selected for testing. In all five instances, the Alexa-trained participants correctly detected that the email is phishing with efficiency ranging between 87.2 and 92.1%. We believe that this result is promising and clearly underlines the potential of any intelligent voice assistants as alternative methods for delivering phishing training.

We also asked all the participants in the study to provide a description on cues they used to spot a potentially phishing email in the vignette. In the case of the phishing email with the authority principle of persuasion, the Alexa-trained participants immediately called the inconsistency between the authority (a university’s financial aid office) and the need for using a login to receive any CARES Act related benefits. Similarly, in the case of the authority/scarcity email, the Alexa-trained participants noticed that a university usually will not sent any email with a “sign in here” link, as well as that it is unlikely there was a scarcity of traffic and therefore a migration was needed.

In the case of the email employing the commitment principle of persuasion, the main rationale for being phished was around the fact that a political campaign communication tried to guilt-trip the receiver in sending money without providing more details on the political platform. In a similar vain, the explanation on why the commitment/liking phishing email stands out, the Alexa-trained participants pointed out that the email plays around the commitment and unequivocal support the particular candidate receives from his supporters. Finally, in the the case of the scarcity/liking phishing email, the rationale provided was that the CDC is hardly to partner with the Bill and Melinda Gates foundation for any report related to COVID-19.

The interaction-based training did not show a statistical significance in the remaining emails from the vignette shown in Table 2. A close inspection of the results indicates that the Alexa-trained participants and the facts-and-advice participants showed better efficiency of over 85.5% in detecting the phishing emails employing the authority/commitment, authority/liking, authority/liking/scarcity, and scarcity. The participants in all three groups were equally good in calling off the phishing emails in the case of social proof, social proof/liking, liking, and scarcity. The facts-and-advice-trained participants were better in calling the phishing email employing the reciprocation, reciprocation/liking, and social proof/commitment.

### Implications for anti-phishing

The results of our study show that the users trust intelligent voice assistants like Alexa in learning more about the threat of phishing emails and the nefarious use of persuasion in communication. Since users personify and highly trust Alexa over a traditional computer or a smartphone, we believe that Alexa can be utilized as a “phishing trainer” on a lager scale. The phishing training can be organized in several forms. For example, the first time a user configures Alexa to manage their emails, Alexa can deliver a quick tutorial with examples on each of the principles of phishing email influences, mentioned in Section 2.5, in a medium “excited” tone [31]. Similarly, an interactive game for phishing training can be developed as an Alexa skill, and we also plan to purse that line of research. Various training emails could be spoken back to users, and based on their answers, Alexa can give explanations in a high “exciting” tone for correct and a medium “disappointed” tone for incorrect decisions. It is worth comparing the impressions of users when given the opportunity to play an interactive game like Jeopardy for the purpose of learning phishing.

From a usable security perspective, a way forward for dealing with suspicious emails is not just to deliver a training for users but also to use Alexa in screening and notifying users about a potentially harmful email in the user’s inbox. For those emails that are suspicious yet anyhow end up in our inbox, email providers like Google Mail provide visual warnings when displaying the email to notify the user that “this message seems dangerous” and offer a button to delete the message right away. A simple adaptation for the Alexa email application is a configuration where Alexa by default reads this warning with a different tone/volume than the usual and directly suggest to the user “It’s probably best that I delete this message right away, okay?” Amazon allows Alexa to express emotions with a different tone, for now only “excited” and “disappointed,” and with high, medium, or low volumes [31]. An update to the Alexa API should allow for “suspicious” tone, but in the meantime a combination of “disappointed” and “high” tones could be used to deliver this *interaction-based phishing warning*.

### Limitations and future work

We utilized a relatively small sample in our study with mostly younger-leaning participants. This limits the generalization of the findings about a more representative population that might have a different approach towards handling phishing training and interacting with Alexa. We did not measure for the frequency of interaction with intelligent voice assistants but studies suggest that the personification and interaction with Alexa is dependent on it [6, 15]. It will be interesting in the future to explore the relationship between one’s susceptibility to phishing and their interaction habits with Alexa that might affect the interactive-phishing training. We used Amazon Alexa in our study, and that might also affect the generalization of the results. Other voice assistants like Google Home, Siri, or Cortana might yield different findings in regard to people’s ability to spot a phishing email. Our study utilized a vignette with 28 emails that were customized based on previous tropes and examples of phishing emails. Different emails from different senders, perhaps including actual fraudulent links, might yield different results. Also, different type of interaction-based training, focused say only on fraudulent URLs and not the principles of persuasion, might yield different results.

## Conclusion

In this work, we set out to understand the effectiveness of an interaction-based phishing training delivered through an intelligent voice assistant—Alexa. This new type of phishing training, we believe, has the potential to help users better understand the threat of phishing in addition to any facts-and-advice training, phishing story, or a phishing game. We tested this interaction-based phishing training and compared with a phishing assessment between three groups of participants (120 total), one that received no training, one that was given a facts-and-advice training, and one that interacted with Alexa to learn about the persuasive nature of the phishing emails. We found that the Alexa-trained participants significantly outperformed the other groups when spotting phishing emails employing the persuasion principles (and or combinations) of authority, authority/scarcity, commitment, commitment/liking, and scarcity/liking. These findings are along the lines of the other studies that tested the effectiveness of traditional forms of phishing training like facts-and-advice, stories, and phishing games. In response, we proposed a couple of solutions for phishing warnings and extended training delivered through Alexa that we plan to pursue in our future research. We hope our results inform the security community about the potential of using Alexa as a phishing teacher.

## Data Availability

The study data is confidential and could be made available upon direct request to the authors.

## Abbreviations

API: Applicaiton Programming Interface
CARES Act: Coronavirus Aid, Relief, and Economic Security Act
CDC: Centers for Disease Control
COVID-19: Coronavirus disease of 2019
IRB: Institutional Review Board
SeBIS: Security Behaviour Intentions Scale
Q[MYAMP: A] Questions and Answers
URL: Uniform Resource Locator
